# Local Wisdom and Diversity of Medicinal Plants in Cha Miang Forest in Mae Kampong Village, Chiang Mai, Thailand, and Their Potential for Use as Osteoprotective Products

**DOI:** 10.3390/plants11111492

**Published:** 2022-06-01

**Authors:** Treethip Sukkho, Chartchai Khanongnuch, Saisamorn Lumyong, Jetsada Ruangsuriya, Thanawat Pattananandecha, Sutasinee Apichai, Fumihiko Ogata, Naohito Kawasaki, Chalermpong Saenjum

**Affiliations:** 1 Department of Biotechnology, Graduate School, Chiang Mai University, Chiang Mai 50200, Thailand; treethip.sk@gmail.com; 2 Center of Excellence for Innovation in Analytical Science and Technology for Biodiversity-Based Economic and Society (I-ANALY-S-T_B.BES-CMU), Chiang Mai University, Chiang Mai 50200, Thailand; thanawat.pdecha@gmail.com (T.P.); sutasinee.apichai@gmail.com (S.A.); 3 Division of Biotechnology, School of Agro-Industry, Faculty of Agro-Industry, Chiang Mai University, Chiang Mai 50100, Thailand; ck_biot@yahoo.com; 4 Research Center for Multidisciplinary Approaches to Miang, Science and Technology Research Institute (STRI), Chiang Mai University, Chiang Mai 50200, Thailand; 5 Department of Biology, Faculty of Sciences, Chiang Mai University, Chiang Mai 50200, Thailand; scboi009@gmail.com; 6 Research Center of Microbial Diversity and Sustainable Utilization, Faculty of Science, Chiang Mai University, Chiang Mai 50200, Thailand; 7 Department of Biochemistry, Faculty of Medicine, Chiang Mai University, Chiang Mai 50200, Thailand; jetsada.ruang@cmu.ac.th; 8 Department of Pharmaceutical Sciences, Faculty of Pharmacy, Chiang Mai University, Chiang Mai 50200, Thailand; 9 Faculty of Pharmacy, Kindai University, 3-4-1 Kowakae, Higashi-Osaka 577-8502, Japan; ogata@phar.kindai.ac.jp (F.O.); kawasaki@phar.kindai.ac.jp (N.K.); 10 Antiaging Center, Kindai University, 3-4-1 Kowakae, Higashi-Osaka 577-8502, Japan

**Keywords:** medicinal plants, “People-Forest-Miang” communities, Cha Miang forest, Mae Kampong Village, osteoprotective products, alkaline phosphatase activity

## Abstract

“People-Forest-Miang” communities are villages located in the cultivated area of *Camellia sinensis* var. *assamica*, or Cha Miang, in northern Thailand. Cha Miang forests are a form of agriculture relying on forest-rich bioresources. This study focuses on a survey of the diversity of medicinal plants used by “People-Forest-Miang” communities in Mae Kampong Village, Chiang Mai, Thailand. The results demonstrated that 73 species of medicinal plants were used to prevent and treat various ailments. The highest number of species (30.14%) was used for musculoskeletal system disorders, followed by digestive system disorders (21.92%) and unspecified medicinal disorders (15.07%). The alkaline phosphatase (ALP) is the most widely recognized biochemical marker for osteoblast activity. The ALP activity of ethanol and deionized water extracts of the nine selected medicinal plants used for musculoskeletal system disorders were examined in the MG63 cell line. The results showed that the numerous water extracts, including MKP1, MKP2, MKP5, MKP6, MKP7, MKP8, and MKP9, and the ethanolic extracts—namely, MKP2, MKP3, MKP7, and MKP9—significantly increased ALP activity in the MG-63 cell line. The findings indicate that some medicinal plants may be further studied for active chemicals and developed as natural active pharmaceutical ingredients for osteoprotective products.

## 1. Introduction

Traditional medicine (TM) comprises the knowledge of indigenous or folk medicine, skills, and practices based on the theories, beliefs, and experiences of native peoples of various cultures; it is used for healthcare and the prevention, diagnosis, improvement, and treatment of different diseases using traditional plant-, animal-, and mineral-based drugs [1]. Historical accounts of TM depict that different plants were used as early as 5000 to 4000 BC in China, and in 1600 BC by Syrians, Babylonians, Hebrews, and Egyptians [2]. TM is still common in China, India, Japan, Pakistan, Sri Lanka, and Thailand [3]. The World Health Organization (WHO) has reported that approximately 80% of the world’s population in developing countries rely on TM to fulfill their daily health needs.

Plants provide a natural supply of food, fuel, shelter, and medicine for human life. Traditional knowledge of medicinal plants has been collected and transmitted from generation to generation through verbal teaching and local practitioners [4]. Some new types of medicine from medicinal plants have traditionally relied on ethnobotanical knowledge. Many common traditional medications have been discovered using ethnobotanical data because most medicinal plants are a major source of pharmaceutical medications, used to treat and cure a variety of ailments and diseases [5]. Natural products, particularly those derived from medicinal plants, are becoming increasingly popular for treating a variety of ailments. Because of their low side effects and resistance, they are becoming more important than synthetic medications [6]. Nowadays, many people from several countries are increasingly turning to TM to help maintain their health, because it is effective, accessible, and has low toxicity and few side effects. Although modern medicine has spread widely in developed countries, TM is still recognized as the primary healthcare system in various rural communities, especially in “People-Forest-Miang” communities where numerous local medicinal plants are used to prevent and cure various ailments experienced in daily life. “People-Forest-Miang” communities are inhabited villages in the area where Assam tea (*Camellia sinensis* var. *assamica*) or Cha Miang in Thai, is naturally cultivated, with high productivity, in highland and mountainous areas in northern Thailand, including the Chiang Mai, Nan, Lampang, Phrae, Phayao, Chiang Rai, and Mae Hong Son Provinces [7]. Assam tea leaves are frequently utilized as a raw material by local northern Thais to make traditional fermented products known as “Miang”, which are chewed during work hours, in cultural ceremonies, and as a welcome household snack [8]. Cha Miang forests are commonly found in watershed areas, classified as 1A watersheds, and are legally designated as protected forest areas [7]. Assam tea cultivation is an example of agroforestry that thrives under the forest canopy’s shade and grows as part of the forest. This local wisdom protects the forest from human encroachment. Therefore, Cha Miang forests are believed to serve as one strategy by which to protect water sources; environmental and natural resources; and the valuable diversity of plants, animals, and fungi [9,10]. It was found that 30.8% of medicinal plants have been used for medicinal purposes. Some of these are bought from shops or traditional practitioners, and the materials are collected from home gardens or Cha Miang forests. Currently, “People-Forest-Miang” communities also use medicinal plants to prevent, treat, and manage various ailments; the communities collect the plants from the Cha Miang forests around the villages and use these based on the knowledge inherited from their ancestors [11].

Thailand has become an “aging society”, which has been ongoing since 2005. In 2022, the population aged 60 years and totaled over 12 million people, or 18.3% of the total population, and is expected to reach 17 million in 2030, accounting for 26.56% of the total population [12,13]. Older individuals often experience many health challenges. The first health problem among older adults is related to physical movement. These diseases are classified as age-related diseases, such as osteoporosis, which is one cause of such problems. Hip fractures among older individuals are associated with osteoporosis, greatly affecting older people’s lifestyles due to limited movement, causing complications and increased mortality. The incidence is increasing worldwide, including in Thailand [13,14,15]. Osteoporosis is caused by an unbalanced rate of bone formation and resorption. Some conditions may result in abnormal bone formation and resorption, such as those in postmenopausal women, those with decreased female hormones, or in men over 65. The estrogen hormone regulates the viability of osteoclast cells involved in bone resorption. When estrogen is lacking, osteoclast cells live longer and increase their activity. As a result, more bone resorption than bone formation occurs, causing the bones to have a porous appearance similar to that of a sponge, and to decrease in bone density and strength; these cause breakages [16,17]. The incidence of fractures among patients aged 65 years in Chiang Mai, Thailand, has increased by 100% (from 600:100,000 to 1200:100,000). The mortality rate after hip fracture within one year is approximately 20%, and death within three years is approximately 50%. In addition, osteoporosis and hip fracture treatment incur a high cost of around THB 120,000 yearly, and few patients can afford to access high-cost treatments [15,18]. In addition, Thailand has been interested in various medicinal plants found in northern Thailand, for example, *Perilla frutescens* (L.) Britton, *Cissus quadrangularis* L., and *Pueraria mirifica* Airy Shaw & Suvat. Numerous studies have shown that these medicinal plants affect bone promotion. For instance, The ethyl acetate fraction of *Perilla* seed meal extract has been proven to have the highest antioxidant and anti-inflammatory activity associated with anti-osteoporotic effects [19]. Furthermore, *P. mirifica* tuberous root extract could improve bone mineral density, bone mineral content, and bone geometry in postmenopausal osteoporotic monkeys [20]. Moreover, the ethanolic fraction of *Cissus quadrangularis* significantly enhanced the alkaline phosphatase (ALP) activity of MG-63 cells by significantly activating the molecules involved in bone formation, such as osteocalcin (OC) and osteoprotegerin (OPG), and reducing the receptor activator of the nuclear factor kappa ligand (RANKL), indicating reduced bone resorption [21]. Alkaline phosphatase is a biochemical marker of bone formation. It is an enzyme that releases inorganic phosphate from phosphoric esters, necessary for mineralization and important for bone formation. This enzyme can be found in the bones, liver, kidneys, intestines, and placenta, but has the highest concentration in bone and liver cells. Serum ALP measurement is important in the diagnosis of liver, bile duct, and bone diseases. In general, having high ALP means liver damage or a condition that increases bone cell activity. ALP binds to the outside of osteoblasts with glycosylphosphatidylinositol. When released from the cell membrane, it is found in the bone matrix. The ALP enzyme from the bone is an important controller in the matrix mineralization process, breaking down inorganicpyrophosphate, which inhibits the matrix mineralization process. In addition, ALP from the bone also helps inorganic phosphate (from pyrophosphate and organic phosphomonoester) in the formation of hydroxyapatite crystals, an inorganic substance that is a component of bones [22,23,24].

The health effects of osteoporosis and the financial barriers to accessing healthcare, as well as the poor documentation of systems of local knowledge of medicinal plants in “People-Forest-Miang” communities, have been described previously. Considering Thailand’s osteoporosis situation and the high cost of treatment, finding treatments using medicinal plants is an alternative treatment option for osteoporosis. Therefore, it is necessary to research medicinal plants that have the potential to treat osteoporosis based on local knowledge. This study focused on surveying the diversity of medicinal plants used by “People-Forest-Miang” communities in Mae Kampong Village, Chiang Mai, Thailand, particularly with regard to bone-related disease treatment. The purpose of this study was to conserve and prepare indigenous medicinal plants in the Cha Miang forest as natural active pharmaceutical ingredients (NAPIs). The selected medicinal plants were also extracted and investigated concerning their cytotoxic activity and osteoporotic protection in a cell-based study through the effect of ALP activity. Moreover, combining local wisdom and advanced knowledge would be useful for further research in pharmacologic studies and potential NAPIs development and preparation. Moreover, the sustainable management of these traditional medicinal plant resources and their protection from extinction are deemed necessary and significant.

## 2. Results

### 2.1. Medicinal Plant Species Diversity

**Figure 1:**
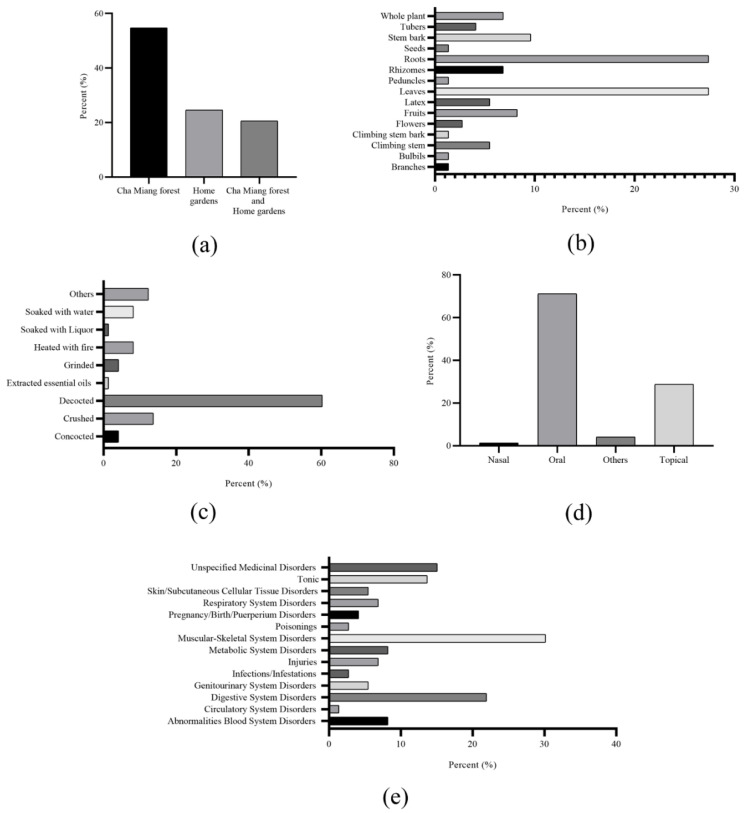
(**a**) Medicinal plants’ habitats; (**b**) percentage of plant parts used from medicinal plant species; (**c**) percentage of medicinal plant preparation methods; (**d**) percentage of administration routes; (**e**) percentage of plant species used as ethnomedicine for a particular ailment.

**Figure 2:**
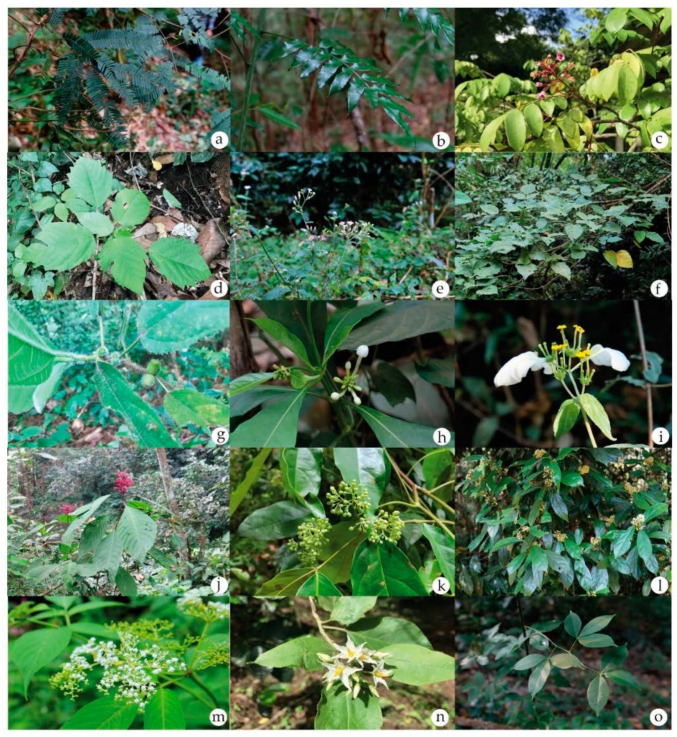
Some diverse medicinal plants from the “People-Forest-Miang” communities of Mae Kampong Village: (**a**) *Albizia myriophylla* Benth.; (**b**) *Angiopteris evecta* (G. Forst.) Hoffm.; (**c**) *Averrhoa carambola* L.; (**d**) *Baliospermum calycinum* Müll. Arg.; (**e**) *Cyanthillium cinereum* (L.) H.Rob.; (**f**) *Erythrina stricta* Roxb.; (**g**) *Ficus hirta* Vahl; (**h**) *Morinda angustifolia* Roxb. var. *angustifolia*; (**i**) *Mussaenda sanderiana* Ridl.; (**j**) *Phlogacanthus curviflorus* (Wall.) Nees; (**k**) *Picrasma javanica* Blume; (**l**) *Pittosporopsis kerrii* Craib; (**m**) *Sambucus javanica* Reinw. ex Blume subsp. *javanica*; (**n**) *Solanum torvum* Sw.; (**o**) *Toddalia asiatica* (L.) Lam.

The traditional practitioners reported a total of 73 medicinal plant species used to prevent and treat various ailments in the study area. The surveyed medicinal plants were distributed in 66 genera and 46 families. Ethnomedicinal information for each species, including family name, scientific name, local name, the part used in preparation, and administration routes, are illustrated in Table 1 and Table 2. The family Euphorbiaceae had the highest proportion of medicinal plants used in comparison to the other families. Together, 24.66% of the medicinal plants were cultivated in home gardens, while 20.55% were found in both home gardens and Cha Miang forests, as shown in Figure 1a. Some diverse medicinal plants from the “People-Forest-Miang” communities of Mae Kampong Village are shown in Figure 2.

#### 2.1.1. Plant Parts Used

Leaves and roots were the most frequently used parts for medicinal purposes in 27.47% of approximately 20 species, followed by stem bark (7 species, 9.59%) and fruits (6 species, 8.22%) (Figure 1b).

#### 2.1.2. Preparation and Route of Administration

Various methods were used to prepare herbal extracts from medicinal plants. The decoction technique (60.27%) was the most common and frequent method of traditional preparation for 44 species, followed by crushing (10 species, 13.70%) and others (9 species, 12.33%), as shown in Figure 1c. Moreover, the oral route was the main route of administration (52 species, 71.23%), followed by the topical route (21 species, 28.77%), and others (3 species, 4.11%), as shown in Figure 1d.

#### 2.1.3. Ethnomedicine Used

Figure 1e shows the percentage of medicinal plants used to treat various ailments, categorized into 14 ailment categories. The highest number of species (22 species, 30.14%) was used for musculoskeletal system disorders, followed by digestive system disorders (6 species, 21.92%) and unspecified medicinal disorders (11 species, 15.07%).

##### Musculoskeletal System Disorders

Musculoskeletal system disorders included bone fractures, bruises, and muscle pain. Approximately 30.14% of the surveyed medicinal plants were used for musculoskeletal-system disorder symptoms. For example, *Sambucus javanica* Reinw. ex Blume (leaves, crushed and pounded) was used for bone fractures, bruises, and muscle pain, and *Zingiber montanum* (J. Koenig) Link ex A. Dietr. (rhizomes, crushed) was used for bone fractures, bruises, and muscle pain.

##### Digestive System Disorders

Digestive system disorders are common symptoms in everyday life, and include abdominal pain, indigestion, diarrhea, food poisoning, and gastritis. *Acorus calamus* L. (the whole plant, decocted), *Zingiber montanum* (J. Koenig) Link ex A. Dietr. (rhizomes, decocted), *Dregea volubilis* (L.f.) Benth. ex Hook.f. (climbing stem, decocted), and *Oroxylum indicum* (L.) Kurz (stem bark, decocted) were used as laxatives to relieve indigestion, constipation, and abdominal pain. *Diospyros glandulosa* Lace (ripe fruits), *Osbeckia stellata* Buch.-Ham. ex Ker Gawl. (roots, decoction), and *Rubus alceifolius* Poir. (roots, decoction) were used as an anti-diarrhea remedy.

##### Tonic

The Mae Kampong villagers’ main occupations are in the agricultural sector, especially in Cha Miang plantations and Miang production, which consumes much energy. Therefore, numerous surveyed medicinal plants were used to maintain energy and nourishment and reduce fatigue symptoms. A single medicinal plant might be used, or a combined formulation might also be used. The widespread methods of preparation were decoction and liquor infusions. The medicinal plants frequently used were *Elephantopus* *scaber* L. var. *scaber* (roots, decoction), *Tacca chantrieri* André and *Tacca integrifolia* Ker Gawl. (tubers, decoction), and *Betula alnoides* Buch.-Ham. ex D.Don (stem bark, soaked with liquor).

##### Respiratory System Disorders

Respiratory system disorders have common symptoms: sneezing and runny nose, nasal congestion, coughing, and sore throat found during certain seasons and weather changes. *Roureopsis stenopetala* (Griff.) G. Schellenb. (consumed fresh fruit) and *Albizia myriophylla* Benth. (ground and mixed with other ingredients) were used for cough relief. Additionally, a decoction of *Schima wallichii* Choisy was used to relieve asthma-like symptoms such as breathing difficulty, tight chest, and coughing caused by swelling of the breathing tubes that carry air in and out of the lungs.

##### Genito-Urinary System Disorders

Most of these drugs were used to treat gallstone disease. Its symptoms depend on the size and number of the stones and where the gallstone is blocked. Symptoms include pain in the waist, abdominal pain, and dysuria. The most common preparation method is a decoction, which is believed to help dissolve the stones. A single plant or a combination might be used. Plants used to treat gallstones include *Baliospermum calycinum* Müll. Arg. (roots, decocted), *Vitex trifolia* L. (roots, decocted), *Averrhoa carambola* L., and *Mussaenda sanderiana* Ridl. (roots, decocted). Moreover, other conditions such as hemorrhoids are caused by swollen veins in the lowest part of the rectum and anus, which are the most common causes of rectal bleeding. Many kinds of plants were used to treat hemorrhoids, such as *Baliospermum calycinum* Müll. Arg. (roots, decocted), *Excoecaria cochinchinensis* Lour. var. *cochinchinensis* (leaves, decocted), and *Polygonum chinense* L. var. *paradoxum* (H. Lév.) A. J. Li (the whole plant, decocted).

##### Pregnancy/Birth/Puerperium Disorder

*Iris domestica* (L.) Goldblatt & Mabb. (rhizomes, ground with water) was used to treat symptoms after delivering a child, such as dizziness, nausea, vomiting, palpitations, fainting, body aches, and pain. The decoction of *Ficus auriculata* Lour. Peduncle was used to induce lactation. Furthermore, *Cephalostachyum virgatum* (Munro) Kurz (leaves, decocted) was used to treat postpartum hemorrhage.

##### Injuries

Mae Kampong community members used *Chromolaena odorata* (L.) R. M. King & H. Rob. (leaves, crushed and pounded) to control and stop bleeding. Then, the latex of *Baliospermum calycinum* Müll. Arg. or *Jatropha podagrica* Hook. was applied to fresh wounds. Moreover, *Erythrina stricta* Roxb. (stem bark, heated with fire, crushed and pounded) was used for wound healing.

##### Skin/Subcutaneous Cellular Tissue Disorders

Extracts to reduce and relieve itching symptoms used the leaves of *Picrasma javanica* Blume (boiled in water and taken in a bath) or *Clausena excavata* Burm.f. (decocted). Moreover, some local practitioners used *Clinacanthus nutans* (Burm.f.) Lindau (leaves, decocted) and *Drynaria quercifolia* (L.) J. Sm. (rhizomes, crushed and pounded) to treat skin-cancer-like symptoms.

##### Abnormal Blood System Disorders

Some medicinal plants were used to relieve iron-deficiency-like symptoms and as appetite stimulants. Decoctions of *Acorus calamus* L. (whole plant), the climbing stem of *Dregea volubilis* (L.f.) Benth. ex Hook.f, the stem bark of *Oroxylum indicum* (L.) Kurz, and the rhizome of *Angiopteris evecta* (G. Forst.) Hoffm. were drunk to nourish the blood.

##### Blood-Glucose-Lowering Plants

Various surveyed medicinal plants were used to reduce blood sugar levels, including a decoction of *Gynostemma pentaphyllum* (Thunb.) Makino leaves, a concoction of *Tacca chantrieri* André, *Tacca integrifolia* Ker Gawl. leaves and flowers, and a decoction of *Pittosporopsis kerrii* Craib fruits.

##### Infections/Infestations

When the body fights off a bacterial infection, an abscess might appear on or under the skin, or even deep inside the body. The latex from *Alstonia rostrata* can be applied to a skin abscess, resulting in the relief of the skin’s infection-like symptoms, including local pain, tenderness, warmth, and swelling. Regarding cardiovascular system disorders, the results demonstrated that only a decoction of *Cinnamomum iners* Reinw. ex Blume stem bark was used as a cardiotonic to increase the heart muscles’ efficiency and contractions, leading to improved blood flow to all body tissues.

##### Unspecified Medicinal Disorders

Unspecified medicinal disorders included mouth ulcers, migraine headaches, hangovers, and erectile dysfunction. Various medicinal plants were used to relieve mouth ulcer symptoms, such as macerated waters of *Spondias pinnata* (L. f.) Kurz stem bark, *Centella asiatica* (L.) Urb. leaves, and the aerial part of *Scoparia dulcis* L. In addition, a decoction of *Melia azedarach* L. fruits was used to relieve migraine headaches; water soaked with *Phrynium pubinerve* Blume roots was used to reduce hangover symptoms; and a decoction of *Elephantopus scaber* L. var. scaber roots was used to improve impotent sexual function.

##### Detoxification

Food poisoning symptoms such as abdominal pain, vomiting, and diarrhea were treated using many plants, for example, *Ficus hirta* Vahl (roots, decocted) and *Micromelum minutum* Wight & Arn. I (leaves, decocted). 

### 2.2. Percentage Yield of Plant Extracts

Table 3 shows the percentage yield of plant extracts in different solvents: *C. iners* bark, *C. stellatopilosus* branches, *D. cirrhosa* var. *cirrhosa* tubers, *P. curviflorus* leaves, *S. javanica* subsp. *javanica* leaves, *S. simpsonii* leaves, *T. asiatica* climbing stem, *V. sambucinum* var. *tomentosum* leaves, and *Z. montanum* rhizomes, in ethanol and water as solvents. In the ethanolic extract, *S. simpsonii* gave the highest yield (19.34%), while the *T. asiatica* extract gave the lowest yield (5.67%). In the water extract, the *V. sambucinum* var. *tomentosum* extract gave the highest yield (13.77%), while *T. asiatica* gave the lowest yield (3.38%).

### 2.3. ALP Activity of Selected Medicinal Plants

**Figure 3:**
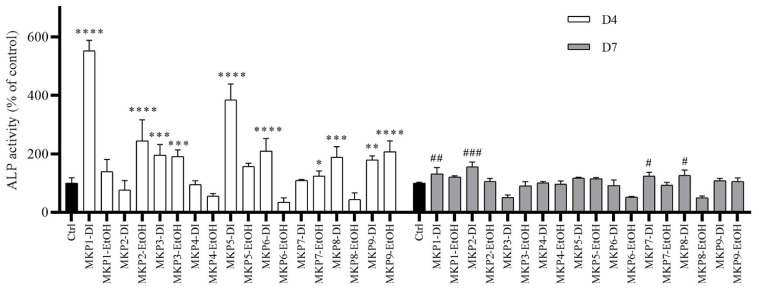
The results show the alkaline phosphatase activity (% of control) of osteoblast-like MG-63 cells after being cultured for 4 and 7 days with different medicinal plant extracts (conc. 25 μg/mL) or only DMEM as control. Data are expressed as mean ± SD where *n* = 3 (significant levels at *, #: *p* < 0.05; **, ##: *p* < 0.01; ***, ###: *p* < 0.001; and ****: *p* < 0.0001, respectively, when compared to control).

The ALP activity measurement was carried out to analyze the effect of nine medicinal plant extracts at a concentration of 25 µg/mL on the osteoblast-like MG63 cell lines. The cytotoxicity of the plant extracts on osteosarcoma MG-63 cell lines was determined using the PrestoBlue assay. The result indicates that the concentration of 25 µg/mL is a suitable concentration because there was no cytotoxicity on the MG-63 cells. The cell viability of the MG-63 cells was tested using the PrestoBlue assay, as shown in Figure S1. The results show that the ALP activity of the MG-63 cells co-cultured with MKP1-DI and MKP8-DI was significantly higher than that of the controls on both day 4 and day 7 of co-incubation (day 4: 552.29 ± 36.28, 188.91 ± 35.50%, and day 7: 131.62 ± 21.63, 126.49 ± 17.96, respectively). Furthermore, the water extracts, including MKP5-DI, MKP6-DI, MKP3-DI, and MKP9-DI, significantly increased ALP activity on day 4—with values of 383.85 ± 54.97, 209.15 ± 42.90, 195.25 ± 36.84, and 179.39 ± 13.59%, respectively, when compared with the control. The ethanolic extracts—namely, MKP2-EtOH, MKP9-EtOH, MKP3-EtOH, and MKP7-EtOH—significantly increased ALP activity on day 4, with values of 244.84 ± 71.41, 207.34 ± 36.87, 191.12 ± 22.26, and 123.97 ± 17.57%, respectively. Moreover, MKP2-DI and MKP7-DI significantly increased ALP activity on day 7, with values of 156.03 ± 15.79 and 123.72 ± 13.36%, respectively, as shown in Figure 3.

## 3. Discussion

Mae Kampong is a village in a valley surrounded by mountains and abundant nature at 950 to 1300 m above sea level. The Mae Kampong villagers have been making “Miang” for a long time. Most of the Cha Miang forest areas are located in the lower montane rainforest, an evergreen forest that is very important to the well-being of the villagers. It serves as a watershed source released into the stream. Lower montane rain forests are found at above average sea levels, at about 1000 to 1900 m. The forest has dense canopies and under growing plants similar to what is seen in tropical rainforests, but it is a dry evergreen forest on lowland and differs in the composition of plant species. Lower montane rainforests consist of temperate and montane species that require relatively cold weather throughout the year. These lowland species represent the dominant flora of tropical and dry evergreen forests. When a lower montane rainforest is destroyed and abandoned for a long time, it will become a secondary evergreen forest such as a lower montane pine-oak forest. Trees are commonly found in lower montane rainforests, such as various types of the family Fagaceae including *Castanopsis acuminatissima* (Blume) A.DC. and *C. diversifolia* (Kurz) King ex Hook.f. Other species of plants include *Magnolia rajaniana* (Craib) Figlar, *M. garrettii* (Craib) V.S.Kumar (Magnoliaceae), *Schima wallichii* Choisy (Theaceae), *Cinnamomum tamala* (Buch.-Ham.) T.Nees & Eberm. (Lauraceae), *Aglaia chittagonga* Miq., *Toona ciliata* M.Roem., (Meliaceae), *Sapindus rarak* DC. (Sapindaceae), *Camellia connata* (Craib) Craib, *C. oleifera* C.Abel var. *confusa*, *Pyrenaria diospyricarpa* var. *camelliiflora* (Kurz) S.X. Yang (Theaceae), *Betula alnoides* Buch.-Ham. ex D.Don (Betulaceae), and *Phlogacanthus curvilonus* (Wall.) Nees (Acanthaceae). The survey of the medicinal plants of Mae Kampong Village found many species of plants that are often found in lower montane rainforests, such as *P. curvilonus* (Wall.) Nees (shrub, often located on stream banks of lower montane rainforests), *B. alnoides* Buch.-Ham. ex D.Don (tree, the pioneer species of lower montane rainforests), and *S. rarak* DC. (tree, the fruit of which the villagers soak in water and use to wash instead of detergent) [25].

Traditional Cha Miang tree cultivation is in harmony with the natural ecosystem because Cha Miang trees are usually planted in forests and highlands, providing good yields and quality. Moreover, Cha Miang tree cultivation can be considered an example of good biodiversity conservation. The traditional method of planting Cha Miang trees is by planting seedlings or seeds interspersed between existing large trees. Cha Miang trees do not grow without the shade of a large tree. In the Cha Miang tree cultivation area, the villagers do not select the headwater area. They do not use deforestation methods such as those used in other commercial tea cultivation and monoagriculture. This makes the plant diversity in the cultivated area utterly different from the cultivation of Cha Miang trees. For this reason, the traditional Cha Miang tree cultivation method of the Mae Kampong people involves local wisdom of the area of conservation, and the management of local resources. Therefore, the biodiversity of the Cha Miang forest area is still high, so it can be used to find natural products, especially medicinal plants. This is consistent with the studies of Preechapanya et al.; these various assessed forms of agroforestry systems on highlands in northern Thailand by analyzing the duration of the sustainability of the system’s economic returns and the acceptance of farmers. Cha Miang forests were proven to be the most suitable agroforestry system in highland areas. In particular, the sustainability of the Cha Miang forest ecosystem is relatively permanent, similar to that of a virgin forest. Rare plants are referred to as plants with a small population and are not yet endangered, but are at risk of being vulnerable in the future. Various threat factors exist such as excessive use, habitat destruction, and invasions of alien species, which cause the plant populations to decrease [26].

Rare plants are defined as plants whose population is known by various sources, and most are small in number compared with other plants [25]. The data on threatened plants in Thailand—collected by the Forest Herbarium Department of National Parks, Wildlife, and Plant Conservation of Thailand—found that some medicinal plants from the Cha Miang forest of Mae Kampong Village are indicated as rare species, such as *Clinacanthus nutans* (Burm. F.) Lindau, *Flemingia sootepensis* Craib, *Croton stellatopilosus* H., and *D. cirrhosa* Lour var. *cirrhosa*, and have been identified as rare plants on a global scale. However, these plants are not rare in the northern Thailand provinces. One plant may be rare in one local area, but in another area, it may be widely distributed [27]. In addition, some plants, such as *Dioscorea cirrhosa* Lour var. *cirrhosa*, are listed as rare on a global scale. The surveys found this plant only at one point in the Cha Miang forest nature trail. Some local healers bring this plant to grow in their home garden areas, for propagation and convenience, where they collect it to make ethnomedicine for the treatment of diseases.

The survey showed that the various kinds of medicinal plants in the Cha Miang forest of Mae Kampong Village were similar to those of the research of Srithi et al. [28], who studied the diversity and use of weeds in the Cha Miang forests of northern Thailand (the Mae Kampong, Pang Kued, and Khun Mae Wak communities). Some similar species such as *Centella asiatica* (L.) Urb., *Imperata cylindrica* (L.) Raeusch., and *Selaginella willdenowii* (Desv. ex Poir.) Baker, were suggested as valuable weed species for an alternative source of food and medicine. Preechapanya [7] reported on the biodiversity of the Cha Miang forest by surveying land use in Cha Miang forest at the Mae Ton Luang River Basin, Thep Sadet Sub-District, Doi Saket District, Chiang Mai, Thailand. The research found that the Cha Miang forest had good biological diversity, especially of plants beneficial for living, such as food plants, plants providing wood used in construction, and medicinal plants. Some plants were the same as those in Mae Kampong Village, such as *Averrhoa carambola* L., *Betula alnoides* Buch-Ham. ex D.Don, *Diospyros glandulosa* Lace, *Imperata cylindrica* (L.) *Raeusch*., *Leea indica* (Burm. F.) Merr., *Osbeckia stellata* Buch.-Ham. ex Ker Gawl., *Melia azedarach* L., *Artocarpus heterophyllus* Lam., *Ficus hirta* Vahl, *Mussaenda sanderiana* Ridl., *Sambucus javanica* Reinw. ex Blume subsp. *javanica*, *Solanum torvum* Sw., and *Schima wallichii* Choisy. Some plants were naturally occurring as well as grown in home gardens [7]. Similar to the Mae Kampong villagers, these villagers would bring the medicinal plant from the Cha Miang forest to their home gardens in a residential area for propagation, making them easier to collect and use; these included plants such as *Phlogacanthus curviflorus* (Wall.) Nees, *Sambucus javanica* Reinw. ex Blume subsp. *javanica*, *Oroxylum indicum* (L.) Kurz, *Tacca chantrieri* André, *Tacca integrifolia* Ker Gawl., *Cinnamomum camphora* (L.) J.Presl, *Cinnamomum iners* Reinw. ex Blume., *Mussaenda sanderiana* Ridl., and *Clausena excavata* Burm.f.

The ethnomedicinal survey showed that the most frequently used medicinal plants were for musculoskeletal system disorders related to agricultural occupations, which are the main occupations of the Mae Kampong villagers. Mae Kampong Village is located amid valleys and natural forests, and includes a high diversity of plants suitable for agriculture. Most of the villagers earn their living in agriculture from Assam tea cultivation, farming, and horticulture, constituting labor-intensive occupations. In ancient times, only a small number of energy-saving devices were employed. Thus, human labor resulted in ailments such as muscle pain, muscle weakness, aches, and exhaustion, comprising obstacles to agricultural occupations. Therefore, most hard-working villagers commonly used medicinal plants to relieve pain—especially back pain, muscle weakness, and aches—as well as to maintain the body [29].

Villagers planted Cha Miang trees under the lower montane rainforest canopy using an indigenous agroforestry planting system. In the area of the Cha Miang forest, headwaters are often the source of small streams, and are important to the villagers’ livelihoods [30,31]. The Royal Forest Department and the National Environmental Committee Office of Thailand defined the WSC; moreover, they provided support for headwater forests in class 1A-quality watersheds with still virgin forests that need to be preserved as watershed areas, because their characteristics and properties may suffer from environmental impacts due to land use that changes the land easily and severely. Mae Kampong Village represents sustainable eco-tourism involving the protection of fertile natural and watershed forests. Because the villagers attach great importance to biodiversity, they have created conservation plans, incentivizing local communities and building networks to undertake biodiversity conservation. Additionally, a campaign has been implemented for the sustainable use of biodiversity in existing community forests. Compared with other forms of highland farming, Cha Miang forests do not destroy natural forests, and they harm natural resources to a relatively small extent. Because no forest area is cleared or burnt, this serves as one of the best practices for highland agriculture [28,32]. Cha Miang forest management can help conserve medicinal plant diversity for use in primary healthcare; it can also help to conserve watershed forests, which play an important role in maintaining water quality because watershed forests reduce the amount of precipitation that passes through the stream.

Although the community forests of Mae Kampong Village are places that the community can use for bioresources due to their high biodiversity, biodiversity must be conserved so as to take sustainable advantage of the biodiversity under the Convention on Biological Diversity (CBD). The CBD is an international environmental agreement, with the goal of all countries’ governments being to develop the country without neglecting conservation of the environment or natural resources. There are three main objectives: the conservation of biodiversity, taking advantage of biodiversity in a sustainable way, and sharing the benefits of the equitable and fair use of genetic resources. The CBD convention was created in 1992, and then put into practice. The Nagoya protocol has also been established, which indicates that genetic resources include any substance of a plant, animal, microorganism, or other origin, as well as the products produced by them such as plant extracts, and local wisdom related to genetic resources. When biological resources are used, one must adhere to three key principles: prior informed consent (PIC), which is the stage for requesting permission to use biological resources; mutually agreed terms (MAT), which concerns how the benefits will be shared; and benefit sharing (BS), which is the sharing of the benefits [33]. In this research, permission to use the genetic resources of Mae Kampong Village was requested, with the aim of exploring medicinal plants and local wisdom related to the utilization of medicinal plants in the community. Moreover, this included requesting permission to collect parts of medicinal plants for use in research, with the stipulation that the plants would be used for research purposes only. In the future, if this research is successful and one wants to make commercial products, one must agree to benefit sharing with the source of the biological resources that are used.

Based on the information of community forests from the Royal Forest Department, Thailand, Mae Kampong villagers have a community forest area of 3,752,000 square meters, which was approved as a community forest in 2000 and has continued as such until the present. The Community Forest Act B.E. (2019) indicates that community forests are places that have community-based forest and natural resource management, or joint resource management by the community. These are activities that support and empower people to participate in planting, conservation, management, protection, and rehabilitation to increase forest integrity, as well as allowing people to use resources and products from the forests under a sustainable management system. This is considered one way to preserve forest areas and the integrity of forest ecosystems, and to keep the ecosystem in balance with the efficient use of forest resource benefits [34]. Mae Kampong Village is located in a high elevation area, and the community forest areas are preserved to serve as watersheds and as a source of food, herbs, and rituals. The villagers also rely on water sourced from the forests for agriculture, and use the forest products as a supplementary income in addition to farming. In addition, community forests are also a source of beliefs and traditions, which are the foundation of community relations. The role of forests in community survival is long-standing and inseparable from the community itself.

From this research, the villagers can obtain the correct scientific information about the medicinal plants used and disseminate this information to the Mae Kampong villagers and tourists. This is considered a combination of the knowledge of medicinal plants that should not be lost over time. Due to most practitioners being very old, the new generation of youth have not inherited this knowledge. Therefore, the collection of knowledge on the utilization of plants is important, and this information can be displayed at the “People-Forest-Miang” museum, which is a museum that tells the story and describes the way of life of the Mae Kampong people for tourists. In addition, the data from the medicinal plant survey can be used as part of the nature walk activities in the nature study trail in the community forest. Because Mae Kampong Village is an eco-tourism village, these activities play an important role in promoting learning about nature and as a tool to convey basic knowledge about the natural environment to people who visit the area. Furthermore, the knowledge gained from this study can also be used as an important database in conservation planning and management, and can also prompt people in the community to realize the value of medicinal plants in their village. At present, there are still local practitioners in the Mae Kampong community using herbal medicines to treat various ailments and propagating medicinal plants for sale to those interested. Therefore, the information obtained from this research can also be used to confirm the properties of these plants as having the potential to treat various diseases, which leads to the promotion of planting in the case of economic crops. This is another way to generate income for families and the community.

After selecting nine medicinal plant species used for bone-related disease treatment, their potential for osteoporotic protection in cell-based studies was investigated. The activity of ALP was evaluated as an indicator of initial osteoblastic differentiation. The ALP activity investigation found that eight medicinal plants had greater osteoblast-promoting activity than that of the control group. Both *S. javanica* subsp. *javanica* (MKP1-DI) and *S. simpsonii* (MKP8-DI) treatment showed a stimulatory effect on human osteoblast-like cells (MG63), with a significant increase in ALP activity as compared with the untreated controls after 4 and 7 days of culture. However, for these two species, information remains lacking about important substances that promote bone cell growth. For centuries, numerous *Sambucus* species have been widely utilized in traditional Chinese medicine to treat bone and joint disorders. Several studies have indicated that plants in the *Sambucus* species have the potential to promote bone cell growth [35,36,37]. *S. williamsii*, as one of the important *Sambucus* species, has been used in Chinese medicinal remedies to treat bone fractures [38]. *S. williamsii* stem extract shows significant bone-protective activity by stimulating the bone formation process and inhibiting bone resorption in both ovariectomized rats and mice, by modulating OPG and RANKL expression [39,40,41]. Moreover, lignans and other chemicals such as phenolic acids and triterpenoids in the ethanol extract of *S. williamsii* stem stimulated osteogenesis by promoting osteoblastic proliferation [39,42]. Several studies have indicated that lignans in the *Sambucus* species are active compounds with potential osteoprotective effects. Xiao et al. [41], who reported several polar constituent compounds of lignans from *S. williamsii*, found that *S. williamsii* significantly promoted cell proliferation via (7R,8R,8′R)-4′-guaiacylglyceryl-Evofolin B, samwinol, and samsesquinoside.

The *Zingiber montanum* (MKP2) rhizome has been widely used among Thai traditional medicine practitioners. Rhizomes contain monoterpenes and terpinen-4-ol, which are typically used in folk medicine to treat inflammation, muscular and joint pain, wounds, and skin diseases [43,44]. An important *Zingiber* species is Ginger (*Zingiber officinale*), for which several research studies have investigated anti-inflammation related bone resorption in arthritis and osteoporosis. Therefore, many studies in a rat model found the active chemical such as polyphenols (gingerols) extracted from the rhizomes of ginger that are bone-protective and prevent bone mineral density loss. This compound was also found to stimulate osteoblast differentiation and increase the transcription levels of osteogenic markers [44,45].

No previous study has investigated the effect of *Cinnamomium iners* (MKP5) on osteoblastic bone formation. Nevertheless, several studies on other *Cinnamomum* species reported that the ethanol extract from the bark of *C. cassia* produced a direct stimulatory effect on bone formation by significantly increasing cell survival, ALP activity, collagen synthesis, and osteocalcin secretion in MC3T3-E1 cells. In addition, The active components of *C. verum*, including cinnamaldehyde and 2-methoxycinnamaldehyde, inhibited the formation of osteoclasts induced with RANKL from RAW 264.7 cells, and inhibited NFATc1 expression, while 2-methoxycinnamaldehyde exhibited remarkable inhibitory effects on the bone resorption of osteoclasts [46].

In the case of *Dioscorea cirrhosa* var. *cirrhosa* (MKP6), the active compound affecting bone formation has not been reported. However, certain research on *Dioscorea* species found that both root and bark extracts of *D*. *batatas* increased osteoblast proliferation and differentiation by stimulating bone matrix maturation and increasing the collagen synthesis, ALP activity, and matrix mineralization of MC3T3E1 cells [47].

Regarding *Viburnum sambucinum* var. *tomentosum* (MKP7), few studies have been conducted on the osteoprotective effects of the important substances of this plant species. Several studies indicated that *Viburnum* species have the potential to promote bone cell growth, such as the fresh fruit juice of *V*. *opulus* as a rich source of phenolic compounds with chlorogenic acid, proanthocyanidins, and catechins. Some phenolic compounds with substantial potential benefits have been shown to affect osteogenic differentiation. In addition, *V*. *opulus* purified phenolic extract was found to increase ALP activity, increase mineralization, and decrease RANKL, preventing its binding with the receptor present in osteoclasts affecting the bone formation process of human osteogenic Saos-2 cells [48,49].

*Toddalia asiatica* (L.) Lam. (MKP9) contains toddaculin, an active compound of *T*. *asiatica* stem bark, affecting both the inhibition of bone resorption and enhancement of bone formation. Toddaculin was found to inhibit the differentiation of osteoclasts via activation of the NF-κB, ERK 1/2 and p38 MAPK signaling pathways in pre-osteoclastic RAW 264 cells, and induced differentiation mineralization in osteoblasts by regulating differentiation factors in pre-osteoblastic MC3T3-E1 cells [50].

Although *Croton stellatopilosus* (MKP3) remains in the Euphorbiaceae family, and is among the most important families containing toxic plants, according to the results of a cytotoxicity test using the PrestoBlue assay, it was found that a concentration of 25–200 µg/mL of *C. stellatopilosus* extract has no cytotoxicity on MG-63 cells, as shown in Figure S1. *C. stellatopilosus* is a popular traditional medicinal plant in Thailand. “Plaunotol” is an active constituent isolated from the leaves of *C. stellatopilosus* with highly effective anti-gastric ulcer properties [51,52]. A related study reported that purified plaunotol extract significantly increased ALP levels and osteoblast activity [53].

This study evaluated the efficacy of eight medicinal plant extracts gathered from a Cha Miang forest and the “People-Forest-Miang” community at Mae Kampong Village, by estimating the ALP activity on osteogenic differentiation ability. Pharmacologic studies have focused on crude extracts, but information regarding the individual bioactive compounds related to anti-osteoporosis activity in some medicinal plants has yet to be elucidated. Importantly, information on the phytochemistry and pharmacology of potential medicinal plants related to biological activities, including *S. javanica* subsp. *javanica* and *S. simpsonii*, should be further studied.

## 4. Materials and Methods

### 4.1. Study Area

**Figure 4:**
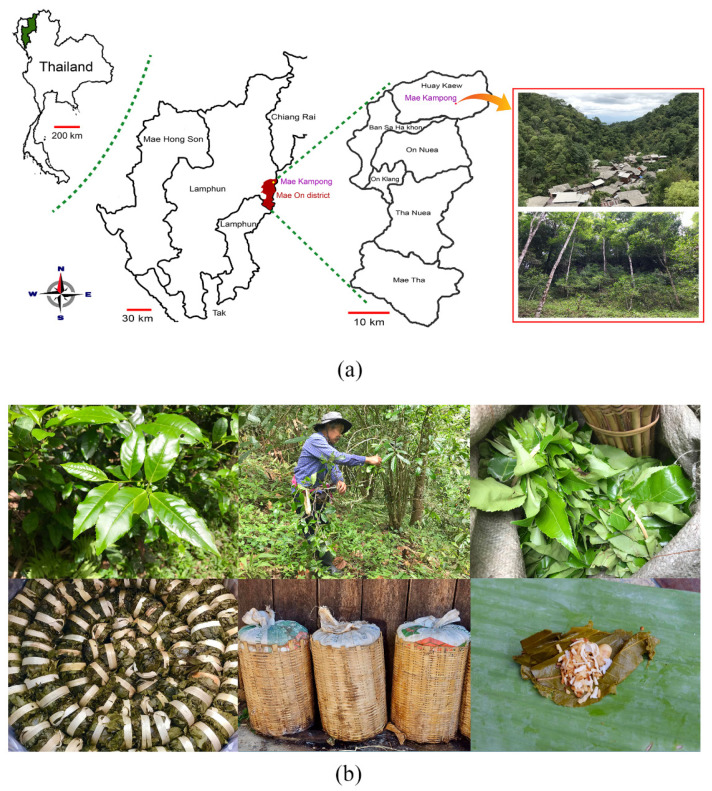
(**a**) Map of Mae Kampong Village, Huay Keaw Sub-District, Mae On District, Chiang Mai province, Thailand; (**b**) Cha Miang leaves (**upper**) and traditional fermented Assam tea leaves, or “Miang” (**lower**).

An ethnobotanical survey of medicinal plants was conducted in the Mae Kampong Village, a small village in a mountainous area in Huay Keaw Sub-District, Mae On District, Chiang Mai, Thailand, located between 18°51′57.0” N and 99°21′11.3” E, as shown in Figure 4a. The total area of the village is about 6.22 square km. The terrain is generally high, and approximately 90% of the area is mountainous. Plentiful natural resources can be found in streams flowing through the village and in the surrounding forest, and this community has been inhabited for more than 200 years. The first people migrated from Doi Saket District, Chiang Mai, Thailand, to find an area suitable for agriculture and Assam tea plantations. In the past, the Lanna people used the leaves of Assam tea, or Cha Miang, to produce fermented products called “Miang”; these were important to the way of life of the Lanna people, who applied local wisdom in Miang production, and this has been part of Lanna culture for centuries. Cha Miang is a tea grown in highland forests. This type of area is designated as a Cha Miang forest, where Assam tea is cultivated. Cha Miang leaves are harvested, steamed, and fermented to produce ancient northern foods with a specific fragrance that are eaten as snacks, as shown in Figure 4b. Cha Miang forests involve sustainable agriculture, and locations where people, forests, and Cha Miang live together are identified as the “People-Forest-Miang” communities [54,55]. According to the watershed classification (WSC), Cha Miang forests are classified as 1A watersheds, protected forest areas including the headwaters of rivers. These areas are usually at a high elevation, around 1300 m above sea level, and have very steep slopes. They should be under permanent forest cover, whereas most forests are evergreen forests [7,56]. The Cha Miang forest area of Mae Kampong Village overlaps the conservation forest area, resulting in abundant forest resources and diverse plants, animals, and microbes. Cha Miang is cultivated together in the forest and grows well in high valley areas. The weather is not too hot or too shaded, becoming a part of the forest. The villagers look after the forest and Cha Miang forest simultaneously. Moreover, Mae Kampong Village represents community-based and sustainable eco-tourism. Tourism in the village is coupled with natural resource conservation through the issuing of village rules and regulations that help maintain and prevent any destruction of natural resources [55].

### 4.2. Data Collection and Medicinal Plants Survey

**Figure 5:**
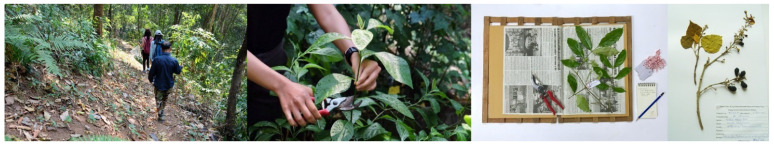
Ethnobotanical plant survey, collection, and preparation of voucher plant specimens.

An ethnobotanical plant survey was conducted in the study area to obtain information on traditional medicinal plants used to prevent and treat various ailments [57]. This study focused on the medicinal plant varieties of “People-Forest-Miang” communities in Mae Kampong Village, where the community and traditional practitioners use the traditional medicinal plants for primary healthcare, to promote health and treat various illnesses. Initially, the survey of medicinal plants was carried out around the villages and Cha Miang forest from April 2019 to March 2020. The key informants were chosen based on traditional knowledge of medicinal plants. In total, 7 key informants (including 1 woman and 6 men with ages ranging from 50 to 79 years) who are traditional practitioners were interviewed. The key informants were interviewed using open-ended and semi-structured questionnaires. Moreover, voucher specimens were collected, pressed, numbered, and dried, as shown in Figure 5. The local names of the plant species, parts used, diseases treated, preparation methods, routes of remedy administration, and the habitats of the medicinal plants were recorded. Morphologic characteristics were examined, photographed, and identified concerning various aspects. For the plant species identification method, we used plant manuals or plant identification keys such as Orders and Families of Malayan Seed Plants, Flora of China, Thai Forest Bulletin (Botany), and Flora of Thailand to determine the plants’ visible characteristics and physical appearance. Identification was undertaken using the relevant literature to compare authentic specimens. The voucher specimens were prepared and deposited in the CMU Herbarium, Faculty of Pharmacy, Chiang Mai University, Thailand.

### 4.3. Chemicals and Reagents

Sodium hydroxide was purchased from Merck (Darmstadt, Germany); ethanol, hydrochloric acid, and dimethylsulphoxide were purchased from RCI Labscan Limited (Bangkok, Thailand); diethanolamine, albumin from bovine serum, CelLytic^TM^ M, penicillin-streptomycin solution, and Bradford reagent were purchased from Sigma-Aldrich Co. (St. Louis, MO, USA); magnesium chloride hexahydrate and 4-nitrophenol were purchased from Sigma-Aldrich Co. (Tokyo, Japan); 4-nitrophenyl phosphate disodium salt hexahydrate was purchased from Sigma-Aldrich, Co. (Dorset, UK); PrestoBlue^TM^ cell viability reagent was purchased from Life Technologies Corporation (Eugene, OR, USA); Dulbecco’s Modified Eagle Medium (DMEM), phosphate-buffered saline pH 7.4, fetal bovine serum (FBS), MEM non-essential amino acid solution, and 0.5% trypsin-EDTA solution were purchased from Life Technologies (Paisley, UK).

### 4.4. Plant Materials

After the medicinal plant survey, the medicinal plant data were grouped into different categories according to body systems. Then, nine medicinal plant species used for bone-related disease treatment were selected for pharmacologic, cytotoxicity, and osteoporotic protection in cell-based studies; the plants included *Sambucus javanica* Reinw. ex Blume subsp. *javanica* (MKP1: leaves), *Zingiber montanum* (J. Koenig) Link ex A. Dietr. (MKP2: rhizomes), *Croton stellatopilosus* H.Ohba (MKP3: branches), *Phlogacanthus curviflorus* (Wall.) Nees (MKP4: leaves), *Cinnamomum iners* Reinw. ex Blume (MKP5: barks), *Dioscorea cirrhosa* Lour var. *cirrhosa* (MKP6: tubers), *Viburnum sambucinum* var. *tomentosum* Hallier f. (MKP7: leaves), *Sambucus simpsonii* Rehder (MKP8: leaves), and *Toddalia asiatica* (L.) Lam. (MKP9: climbing stem). We selected medicinal plants species based on the frequency of use and chose only plants used for bone-related disease treatment and non-harvested plants that were considered rare or as constituting a low population in the northern Thailand provinces; harvested only leaves, bark, branches, tubers, or rhizomes; and avoided picking the root or whole plant. When harvesting, removing only the part to be used and avoiding damage caused by uprooting plant individuals is important to ensure plants can continue growing [58,59,60]. 

### 4.5. Collection and Preparation of Plant Materials

All nine collected medicinal plant parts for bone-related disease treatment were identified. A voucher specimen was prepared and preserved at the Faculty of Pharmacy, Chiang Mai University. The samples were washed and chopped into small pieces and allowed to dry under shade or in a hot-air oven at 50 °C for 12 h. The dried samples were ground into powder using a blender. The plant material powder (100 g) was extracted separately using two different solvents (400 mL)—deionized water (boiled at 100 °C) and 80% ethanol (boiled at 80 °C)—in a water bath for 1 h. This process was repeated three times, and then the mixtures were filtered using filter paper. The solvent was evaporated under reduced pressure and dried under a vacuum to obtain a crude extract. Then, the weight and extraction yields (% yield) were calculated, and these were stored in air-tight containers at −20 °C until used.

### 4.6. Culture of MG-63 Cell Lines and Biochemical Tests

#### 4.6.1. Culture of MG-63 Cell Lines

The human osteosarcoma cell line (MG-63) was purchased from the American Type Culture Collection (ATCC^®^ CRL-1427™, Manassas, VA, USA). The cells were cultured in a 75 cm^2^ flask in complete media, including DMEM, 10% FBS, 1% nonessential amino acids, and 1% penicillin-streptomycin solution under humid conditions in a humidified incubator (New Brunswick^TM^ Galaxy 48 S CO_2_ Incubators, Eppendorf Ag, Hamburg, Germany) at 37 °C and 5% CO_2_. The MG-63 cells were subcultured twice weekly. When the cells reached 70 to 80% confluency, the cells were trypsinized with 0.25% trypsin-EDTA for 5 min. Then, the completed media were added to the inactivated trypsin, and the cells were seeded in a new flask [21].

#### 4.6.2. Determination of ALP Activity

ALP activity was determined using a method adapted from [21]. The cells were trypsinized and plated at 1.0 × 10^4^ cells/cm^2^ (500 µL/well) in each well of 24-well plates. After 24 h incubation at 37 °C in humid conditions containing 5% CO_2_, the cells were treated with medicinal plant extracts at a final concentration of 25 µg/mL with 500 µL/well. The plates were incubated at 37 °C in humid conditions with 5% CO_2_ for 4 and 7 days, and with medium changes at 4-day intervals. Culture medium supernatants were collected, and the cells were lysed to yield cell lysates using celLytic^TM^ M (300 µL/well) and stored at −20 °C for further analyses. ALP activity was determined by the rate of *p*-nitrophenol phosphate (pNPP) conversion to *p*-nitrophenol (pNP). The concentrations of 0.125 to 3 µg/mL of pNP were prepared using 1 M diethanolamine (DEA) and 0.5 mM MgCl_2_·6H_2_O, pH 9.8 as the solvent. Next, 100 μL of different concentrations of pNP were added to each well of the 96-well plates in triplicate. Then, 25 μL of 1 M NaOH 25 µL/well was added before measuring at 405 nm using microplate readers (Spectramax M3, USA). The absorbance of different concentrations was used to establish a standard curve of pNP. Then, 20 μL of the cell lysates was added to 80 μL of the 1 mM pNPP solution (5 replicates). The mixture was mixed well and incubated at 37 °C for 120 min. The reaction was stopped by adding 25 μL of 1 M NaOH solution, and the absorbance was analyzed at 405 nm using microplate readers. Finally, the absorbance was recorded in order to analyze the amount of pNP in the cell lysates by comparison with the pNP standard curve. The ALP activity was expressed in the normalized values of the total protein.

#### 4.6.3. Determination of Total Protein

The amount of protein was measured using the Bradford reagent [21]. The protein produced by the cell lines was analyzed using the Bradford reagent, and bovine serum albumin (BSA) was used as the standard. The concentrations of 5 to 300 µg/mL of BSA were prepared using 1 M diethanolamine (DEA) and 0.5 mM MgCl_2_·6H_2_O, pH 9.8. In total, 20 μL of different concentrations of BSA was added to each well of the 96-well plates in triplicate. Then, 200 μL of Bradford reagent was added and incubated for 5 min at room temperature, and absorbance was measured at 595 nm using microplate readers. The absorbance was used to create a standard curve of BSA. Exactly 20 μL of the cell lysates was added to each well of the 96-well plates in triplicate, and 200 μL of Bradford reagent was added and left to stand for 5 min at room temperature; absorbance was measured at 595 nm using microplate readers. Finally, the absorbance was analyzed as the amount of protein compared with the BSA standard curve.

### 4.7. Statistical Analysis

The data were shown as the mean ± SD of three independent tests. One-way analysis of variance (ANOVA), followed by the Dunnett test, was performed using GraphPad Prism Software, version 8.0 (GraphPad Software, Inc., La Jolla, CA, USA) for statistical analysis. Statistical significance was defined as *p* < 0.05.

## 5. Conclusions

The “People-Forest-Miang” community of Mae Kampong Village use ethnomedicinal knowledge inherited from ancestors and acquired through cumulative experience to heal minor ailments such as stomach ache, diarrhea, indigestion, wounds, fever, and muscle pain. Advances in contemporary medicine now play an essential role in rural communities’ healthcare systems. However, the use of medicinal plants to cure several kinds of diseases remains popular because they contain beneficial properties, produce no side effects, and are inexpensive. Most medicinal plants are collected by local healers skilled in using herbal medicine to treat diseases. They are collected from the Cha Miang forest around village, from farmlands, and in home gardens cultivated by the community. The survey showed that many medicinal plants are used in Mae Kampong Village to treat various ailments. This ethnomedicinal knowledge of medicinal plant use has been accumulated and transferred from generation to generation through oral teaching, but has not been recorded and remains mostly among traditional healers, who are mostly older people. This local wisdom remains poorly documented and is in danger of disappearing. Documenting ethnomedicinal knowledge is clearly needed before it becomes lost to future generations. Moreover, combining local wisdom and advanced knowledge would be useful for further research in pharmacologic and phytochemical studies to confirm the proper validity of these medicines and potential nutraceutical development. In addition, the sustainable use and management of these medicinal plant resources and the conservation of watershed forests are important. The investigation of ALP activity using cell-based in vitro experiments clearly showed that many traditional medicines from the Cha Miang forest of Mae Kham Pong Village presented anti-osteoclastogenic activity and exhibited positive effects on MG-63 cells, significantly enhancing their ALP enzyme activity. Our data showed that some medicinal plants might be valuable anti-osteoporosis agents. These active compounds, with the potential to be used as NAPIs agents, could help prevent osteoporosis and promote health.

## Figures and Tables

**Table 1:** Distribution of medicinal plant species in the Cha Miang forest of Mae Kampong Village.

Families	Number of Species	Families	Number of Species
Euphorbiaceae	5	Iridaceae	1
Asteraceae	4	Juglandaceae	1
Adoxaceae	3	Maranthaceae	1
Apocynaceae	3	Marattiaceae	1
Dioscoreaceae	3	Melastomataceae	1
Fabaceae	3	Meliaceae	1
Lamiaceae	3	Menispermaceae	1
Moraceae	3	Mimosaceae	1
Poaceae	3	Oxalidaceae	1
Rubiaceae	3	Papilionaceae	1
Rutaceae	3	Piperaceae	1
Acanthaceae	2	Plantaginaceae	1
Lauraceae	2	Plumbaginaceae	1
Acoraceae	1	Polygonaceae	1
Amaryllidaceae	1	Polypodiaceae	1
Anacardiaceae	1	Rosaceae	1
Apiaceae	1	Selaginellaceae	1
Betulaceae	1	Simaroubaceae	1
Bignoniaceae	1	Smilacaceae	1
Connaraceae	1	Solanaceae	1
Cucurbitaceae	1	Theaceae	1
Ebenaceae	1	Vitaceae	1
Icacinaceae	1	Zingiberaceae	1
Iridaceae	1		

**Table 2:** Medicinal plant list of Mae Kampong Village, Huay Keaw Sub-District, Mae On District, Chiang Mai, Thailand.

Family	Scientific Name	Local Name	Habitats	Part Used	Disorders Treated	Preparation	Administration Routes	Frequency of Use (%)	Voucher Specimen
Acanthaceae	*Clinacanthus nutans* (Burm.f.) Lindau	Sa Led Pung Porn, Pha Ya Yor	Home gardens	Leaves	Skin cancers, shingles	Decocted	Oral	85.71	0023268
	*Phlogacanthus curviflorus* (Wall.) Nees	Hom Jhang	Cha Miang forest and home gardens	Leaves	Muscle cramp	Crushed	Topical	85.71	0023269
Acoraceae	*Acorus calamus* L.	Hang Khaw	Home gardens	All	Indigestion, constipation, nourish blood	Decocted	Oral	42.86	-
Adoxaceae	*Sambucus javanica* Reinw. ex Blume subsp. *javanica*	Sa Pan Kon	Cha Miang forest and home gardens	Leaves	Bone fractures, bruises, muscle pain Indigestion Constipation	Crushed	Topical	100	0023270
	*Sambucus simpsonii* Rehder	Aoon Ban	Home gardens	Leaves	Bone fractures, bruises, muscle pain	Crushed	Topical	100	0023285
	*Viburnum sambucinum* var. *tomentosum* Hallier f.	Aoon Pa	Cha Miang forest	Leaves	Bone fractures, bruises, muscle pain	Crushed	Topical	85.71	0023314
Amaryllidaceae	*Crinum asiaticum* L.	Phlab phlung	Home gardens	Leaves	Muscle pain Bone fractures, bruises	Heated with fire Crushed	Topical	42.86	-
Anacardiaceae	*Spondias pinnata* (L. f.) Kurz	Kok Thai	Cha Miang forest	Stem bark	Mouth ulcer	Soaked with water	Oral	42.86	-
Apiaceae	*Centella asiatica* (L.) Urb.	Bua Bok	Home gardens	Leaves	Mouth ulcer	Soaked with water	Oral	42.86	-
Apocynaceae	*Alstonia rostrata* C.E.C.Fisch.	Mai Teen Nok	Cha Miang forest	Latex	Abscess	None	Topical	14.29	0023271
	*Alstonia* sp.	Mai Teen Ped	Cha Miang forest	Latex	Abscess	None	Topical	14.29	0023272
	*Dregea volubilis* (L.f.) Benth. ex Hook.f.	Pak Huan	Home gardens	Climbing stem	Indigestion, Constipation, nourish blood	Decocted	Oral	14.29	-
	*Chromolaena odorata* (L.) R. M. King & H. Rob.	Sab Suea	Cha Miang forest	Leaves	Stop the bleeding	Crushed	Topical	100	0023273
Asteraceae	*Cyanthillium cinereum* (L.) H.Rob.	Pa Hiew Mhong	Home gardens	Roots	Cancers	Decocted	Oral	28.57	0023274
	*Eclipta prostrata* (L.) L.	Hom Kiew	Cha Miang forest and home gardens	Leaves	Muscle cramp	Crushed	Topical	71.43	0023275
	*Elephantopus scaber* L. var. *scaber*	Doh Mai Roo Lom	Cha Miang forest	Roots	Tonic Improve sexual performance	Decocted	Oral	100	-
Betulaceae	*Betula alnoides* Buch.-Ham. ex D.Don	Gam Lang Seua Krohng	Cha Miang forest	Stem bark	Muscle pain, bruises Tonic	Extracted essential oils Soaked with Liquor	Topical Oral	100	0023276
Bignoniaceae	*Oroxylum indicum* (L.) Kurz	Lid Mai	Cha Miang forest and home gardens	Stem bark	Indigestion, Constipation, nourish blood	Decocted	Oral	57.14	-
Connaraceae	Roureopsis stenopetala (Griff.) G. Schellenb.	Ma kham Kreua	Cha Miang forest	Fruits	Cough	Consumed fresh fruit	Oral	28.57	0023277
Cucurbitaceae	*Gynostemma pentaphyllum* (Thunb.) Makino	Jiew Ku Laan	Cha Miang forest and home gardens	Leaves	Reduce blood sugar levels	Soaked with hot water	Oral	28.57	0023278
Dioscoreaceae	*Dioscorea cirrhosa* Lour var. *cirrhosa*	Plao Lueat	Cha Miang forest and home gardens	Bulbils Tubers	Anemia Hemorrhoids Back and low back pain	Decocted	Oral	100	0023279
	*Tacca chantrieri* André	Nuem Rue See	Cha Miang forest and home gardens	Tubers Leaves, flower	Tonic Reduce blood sugar levels	Decocted Concocted	Oral	71.43	-
	*Tacca integrifolia* Ker Gawl.	Nuem Rue See	Cha Miang forest and home gardens	Tubers Leaves, flower	Tonic Reduce blood sugar levels	Decocted Concocted	Oral	71.43	-
Ebenaceae	*Diospyros glandulosa* Lace	Ma Kluai rue see	Cha Miang forest	Fruits	Diarrhea	Consumed ripe fruits	Oral	57.14	-
Euphorbiaceae	*Baliospermum calycinum* Müll. Arg.	Plao Tong Taek	Cha Miang forest	Roots Latex	Hemorrhoids Stone disease Cuts, wounds	Decocted Decocted Applied to wounds	Oral Topical	57.14	0023281
	*Croton oblongifolius* Roxb.	Plao Yai	Cha Miang forest and home gardens	Leaves	muscle pain	Heated with fire	Topical	100	0023313
	*Croton stellatopilosus* H.Ohba	Plao Lek	Cha Miang forest and home gardens	Leaves	Muscle pain	Heated with fire	Topical	100	0023282
Euphorbiaceae	*Excoecaria cochinchinensis* Lour. var. *cochinchinensis*	Lin kra Beu	Home gardens	Leaves	Hemorrhoids	Decocted	Oral	28.57	-
	*Jatropha podagrica* Hook. f.	Hanuman Nang Thean	Home gardens	Latex	Cuts, wounds	Applied to cuts	Topical	57.14	-
Fabaceae	*Campylotropis* sp.	Tua Doi	Cha Miang forest	Roots	Back and low back pain, bone pain, knee pain	Decocted	Oral	14.29	0023283
	*Derris* sp.	Kum Lung Wua Tha Leung	Cha Miang forest	Roots	Tonic	Decocted	Oral	14.29	0023290
	*Erythrina stricta* Roxb.	Tong Luang	Cha Miang forest	Stem bark	Cuts, wounds	Heated with fire, crushed	Topical	42.86	0023284
Icacinaceae	*Pittosporopsis kerrii* Craib	Ba Kom	Cha Miang forest	Fruits	Reduce blood sugar levels	Decocted	Oral	28.57	0023286
Iridaceae	*Iris domestica* (L.) Goldblatt & Mabb.	Wan Meed Yub	Cha Miang forest	Rhizomes	Puerperium disorders	Grinded with water	Oral	14.29	-
Juglandaceae	*Engelhardia spicata* Blume	Ka Hot	Cha Miang forest	Stem bark	Toothaches, gingivitis	Decocted and rinsed the mouth	Others	14.29	0023287
Lamiaceae	*Congea tomentosa* Roxb.	Tao Aim On	Cha Miang forest	Roots	Back and low back pain, promote the appetite	Decocted	Oral	14.29	0023288
	*Vitex trifolia* L.	Dok Gum Ber	Home gardens	Roots	Stone disease	Decocted	Oral	28.57	-
	*Vitex peduncularis* Wall. ex Schauer	Ka Sam Peek	Cha Miang forest	Roots	Tonic, back and low back pain	Decocted	Oral	85.71	-
Lauraceae	*Cinnamomum camphora* (L.) J. Presl	Gar Ra Boon	Cha Miang forest and home gardens	Leaves	Muscle pain	Heated with fire	Topical	100	-
Lauraceae	*Cinnamomum iners* Reinw. ex Blume	Aob Choie	Cha Miang forest and home gardens	Stem bark	Cough, Reduce blood sugar levels Nasal polyps Cardiotonic	Grinded and mixed with other ingredients Grinded, wrapped in banana leaves and smoking Decocted	Oral Nasal Oral	100	0023289
Maranthaceae	*Phrynium pubinerve* Blume	Tong Sard	Cha Miang forest	Roots	Cure a hangover	Soaked with water	Oral	28.57	0023280
Marattiaceae	*Angiopteris evecta* (G. Forst.) Hoffm.	Kib Rat	Cha Miang forest	Rhizomes	Nourish blood	Decocted	Oral	14.29	-
Melastomataceae	*Osbeckia stellata* Buch-Ham. ex Ker Gawl.	Ba Ah	Cha Miang forest	Roots	Diarrhea	Decocted	Oral	71.43	0023291
Meliaceae	*Melia azedarach* L.	Mai Hiean	Cha Miang forest	Fruits	Migraine headache	Decocted	Oral	14.29	-
Menispermaceae	*Parabaena sagittata* Miers	Thao Wan Priang	Cha Miang forest	Climbing stem	Back and low back pain, promote the appetite	Decocted	Oral	85.71	0023292
Mimosaceae	*Albizia myriophylla* Benth.	Cha Aim Thed	Cha Miang forest	Climbing stem bark	Diabetes Cough	Grinded and mixed with other ingredients	Oral	14.29	0023293
Moraceae	*Artocarpus heterophyllus* Lam.	Ba Hnun	Home gardens	Roots	Diarrhea	Decocted	Oral	100	-
	*Ficus hirta* Vahl	Duea Din	Cha Miang forest	Roots	Detoxification	Decocted	Oral	71.43	0023294
Moraceae	*Ficus auriculata* Lour.	Ma Duea Pa	Cha Miang forest	Peduncles Fruits	Milk-increasing Diarrhea	Decocted Consumed raw fruit	Oral	71.43	-
Oxalidaceae	*Averrhoa carambola* L.	Ma Feuang	Home gardens	Roots	Stone disease	Decocted	Oral	100	-
Papilionaceae	*Flemingia sootepensis* Craib	Ma Hae Nok	Cha Miang forest	Roots	Back and low back pain,	Decocted	Oral	71.43	0023295
Piperaceae	*Piper* sp.	Ja Kan Din	Cha Miang forest	Climbing stem	Tonic	Decocted	Oral	14.29	0023296
Plantaginaceae	*Scoparia dulcis* L.	Ya Phak Khwai	Home gardens	All	Mouth ulcer	Soaked with water	Oral	14.29	0023297
Plumbaginaceae	*Plumbago indica* L.	Pid Pew Dang	Home gardens	All	Muscle cramp	Crushed	Topical	100	0023298
Poaceae	*Cephalostachyum virgatum* (Munro) Kurz	Phai Here	Cha Miang forest	Stem bark Leaves	Cuts, wounds Postpartum hemorrhage	Scraped Decocted	Topical	28.57	0023299
Poaceae	*Imperata cylindrica* (L.) Raeusch.	Ya Kha	Home gardens and Cha Miang forest	Roots	Mouth ulcer	Soaked with water	Oral	100	-
	*Oryza sativa* L.	Khao	Home gardens	Seeds	Hemorrhoids	Decocted	Oral	28.57	-
Polygonaceae	*Polygonum chinense* L. var. *paradoxum* (H. Lév.) A. J. Li	Phai Jang	Cha Miang forest	All	Hemorrhoids	Decocted	Oral	57.14	0023300
Polypodiaceae	*Drynaria quercifolia* (L.) J. Sm.	Kood Aom	Cha Miang forest	Rhizomes	Skin cancers	Crushed	Topical	28.57	0023301
Rosaceae	*Rubus alceifolius* Poir.	Ma Hooh Luang	Cha Miang forest	Roots	Diarrhea	Decocted	Oral	85.71	0023302
Rubiaceae	*Lasianthus cyanocarpus* Jack	Gum Lung Chang San	Cha Miang forest	Roots	Tonic, back and low back pain	Decocted	Oral	85.71	0023312
Rubiaceae	*Morinda angustifolia* Roxb. var. *angustifolia*	Pha Ya Rak Diew	Cha Miang forest	Roots	Back and low back pain, promote the appetite	Decocted	Oral	100	0023303
	*Mussaenda sanderiana* Ridl.	Dok Pee Seue	Cha Miang forest and home gardens	Roots	Stone disease	Decocted	Oral	57.14	0023304
Rutaceae	*Clausena excavata* Burm.f.	Mai Kee Hao	Cha Miang forest and home gardens	Leaves	Skin disease	Decocted	Topical	28.57	0023305
	*Micromelum minutum* Wight & Arn. I	Mai Mon	Cha Miang forest	Leaves	Detoxification	Boiled in water	Bath	57.14	-
	*Toddalia asiatica* (L.) Lam.	Ma Kra Teub Rong	Cha Miang forest	Climbing stem	Tonic	Decocted	Oral	100	0023306
Selaginellaceae	*Selaginella helferi* Warb.	Ya mung Tao	Cha Miang forest	All	Swelling of hands and feet	Decocted	Oral	28.57	0023307
Simaroubaceae	*Picrasma javanica* Blume	Gom Dum	Cha Miang forest	Leaves	Skin disease	Boiled in water	Bath	100	0023308
Smilacaceae	*Smilax corbularia* Kunth	Hua Khao Yen	Cha Miang forest	Rhizomes	Tonic, promote the appetite	Decocted	Oral	100	0023309
Solanaceae	*Solanum torvum* Sw.	Ba Kuea Puang	Home gardens	Fruits	Eye disease	Concocted	Oral	100	0023310
Theaceae	*Schima wallichii* Choisy	Tha Low	Cha Miang forest	Branches	Asthma	Consumed water from branches	Oral	57.14	-
Vitaceae	*Leea indica* (Burm. f.) Merr.	Kheung Khang Ma	Cha Miang forest	Roots	Tonic	Decocted	Oral	85.71	0023311
Zingiberaceae	*Zingiber montanum* (J. Koenig) Link ex A. Dietr.	Poo Loei	Cha Miang forest	Rhizomes	Bone fractures, bruises, muscle pain Indigestion, constipation, nourish blood	Crushed Decocted	Topical Oral	100	0023315

**Table 3:** Percentage yield of plant extracts in different solvents.

Plant	Parts Used	Percentage Yield of Extract (*w*/*w*%)	
		Ethanol Extract	Water Extract
*Cinnamomum iners* Reinw. ex Blume	Bark	13.39	6.21
*Croton stellatopilosus* H.Ohba	Branches	6.16	4.68
*Dioscorea cirrhosa* Lour var. *cirrhosa*	Tubers	17.03	7.17
*Phlogacanthus curviflorus* (Wall.) Nees	Leaves	11.95	7.03
*Sambucus javanica* Reinw. ex Blume subsp. *javanica*	Leaves	13.15	11.52
*Sambucus simpsonii* Rehder	Leaves	19.34	13.49
*Toddalia asiatica* (L.) Lam.	Climbing stem	5.67	3.38
*Viburnum sambucinum* var. *tomentosum* Hallier f.	Leaves	15.11	13.77
*Zingiber montanum* (J. Koenig) Link ex A. Dietr.	Rhizomes	10.32	5.32

## Supplementary Materials

The following supporting information can be downloaded at: https://www.mdpi.com/article/10.3390/plants11111492/s1, Figure S1: Cell Viability of MG-63 cells by PrestoBlue assay.
