# Supplementary material for: Local Wisdom and Diversity of Medicinal Plants in Cha Miang Forest in Mae Kampong Village, Chiang Mai, Thailand, and Their Potential for Use as Osteoprotective Products
Source: Plants (Basel). 2022 Jun 1;11(11):1492. doi: 10.3390/plants11111492 (PMC9182823; doi:10.3390/plants11111492)
Supplement: Supplementary file 1 [file plants-11-01492-s001.zip › plants-1713536-supplementary.pdf]

plants

Article

Local Wisdom and Diversity of Medicinal Plants in Cha Miang Forest in Mae Kampong Village, Chiang Mai, Thailand, and Their Potential for Use as Osteoprotective Products

Treethip Sukkho [1,2], Chartchai Khanongnuch [3,4], Saisamorn Lumyong [5,6], Jetsada Ruangsuriya [7], Thanawat Pattananandecha [2,8], Sutasinee Apichai [2,8], Fumihiko Ogata [9], Naohito Kawasaki [9,10] and Chalermpong Saenjum [2,8,*]

1 Department of Biotechnology, Graduate School, Chiang Mai University, Chiang Mai 50200, Thailand; treethip.sk@gmail.com
2 Center of Excellence for Innovation in Analytical Science and Technology for Biodiversity-Based Economic and Society (I-ANALY-S-T_B.BES-CMU), Chiang Mai University, Chiang Mai 50200, Thailand; thanawat.pdecha@gmail.com (T.P.); sutasinee.apichai@gmail.com (S.A.)
3 Division of Biotechnology, School of Agro-Industry, Faculty of Agro-Industry, Chiang Mai University, Chiang Mai 50200, Thailand; ck_biot@yahoo.com
4 Research Center for Multidisciplinary Approaches to Miang, Science and Technology Research Institute (STRI), Chiang Mai University, Chiang Mai 50200, Thailand
5 Department of Biology, Faculty of Sciences, Chiang Mai University, Chiang Mai 50200, Thailand; scboi009@gmail.com
6 Research Center of Microbial Diversity and Sustainable Utilization, Faculty of Science, Chaing Mai University, Chiang Mai 50200, Thailand
7 Department of Biochemistry, Faculty of Medicine, Chiang Mai University, Chiang Mai 50200, Thailand; jetsada-ruang@cmu.ac.th
8 Department of Pharmaceutical Sciences, Faculty of Pharmacy, Chiang Mai University, Chiang Mai 50200, Thailand
9 Faculty of Pharmacy, Kindai University, 3-4-1 Kowakae, Higashi-Osaka, Osaka 577-8502, Japan
10 Antiaging Center, Kindai University, 3-4-1 Kowakae, Higashi-Osaka, Osaka 577-8502, Japan ogata@phar.kindai.ac.jp (F.O.); kawasaki@phar.kindai.ac.jp (N.K.)
* Correspondence: chalermpong.saenjum@gmail.com or chalermpong.s@cmu.ac.th; Tel.: +668-9950-4227

Supplementary Materials

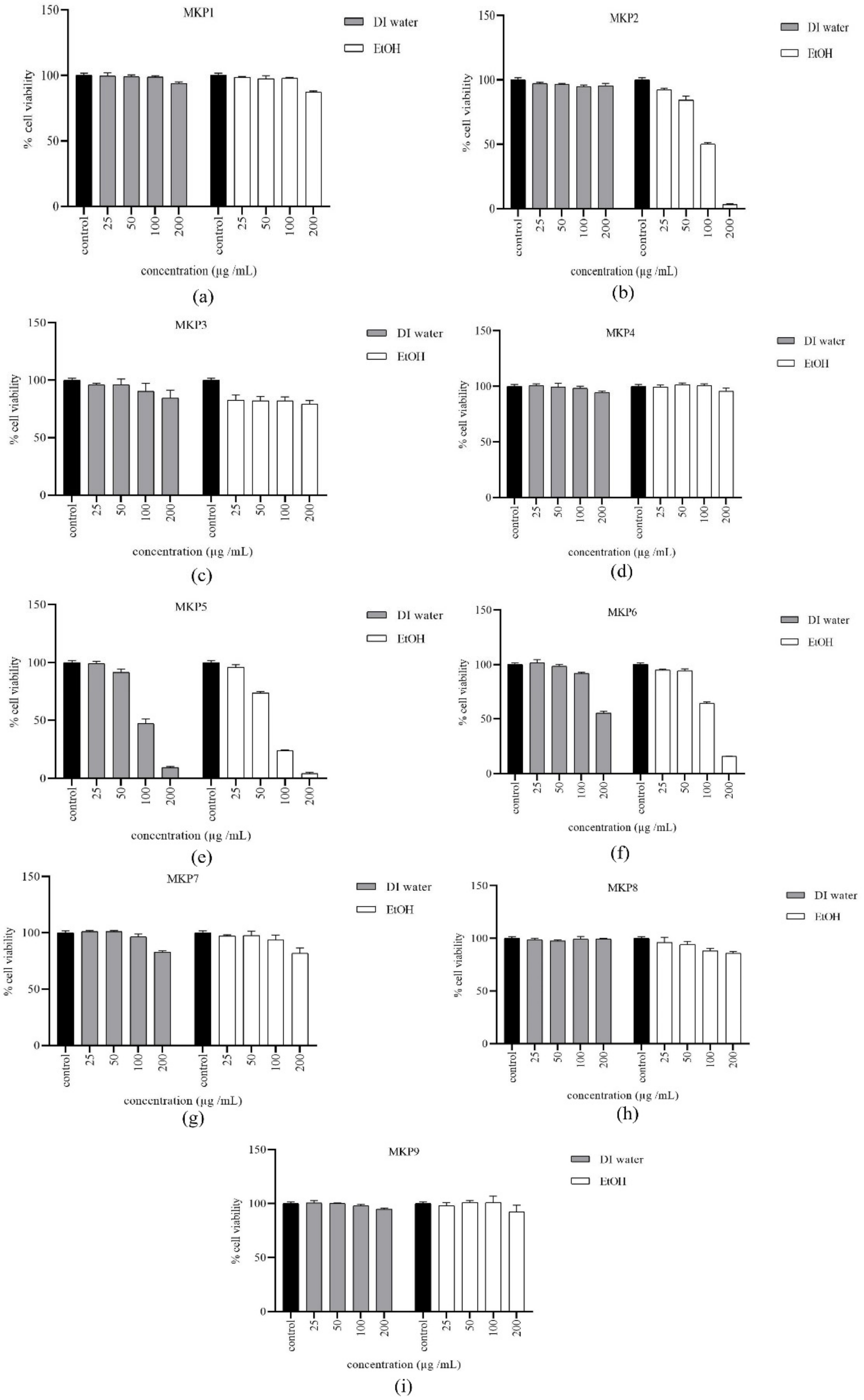

Figure S1. Cell Viability of MG-63 cells by PrestoBlue assay. (a) S. *javanica* subsp. *javanica* (MKP1), (b) *Z. montanum* (MKP2), (c) *C. sublyratus* (MKP3), (d) *P. curviflorus* (MKP4), (e) *C. iners* . (MKP5), (f) *D. cirrhosa* var. *cirrhosa* (MKP6), (g) *V. sambucinum* (MKP7), (h) *S. simpsonii* (MKP8) and (i) *T. asiatica* (MKP9).
